# Real-Time Fall Risk Assessment Using Functional Reach Test

**DOI:** 10.1155/2017/2042974

**Published:** 2017-01-10

**Authors:** Brian Williams, Brandon Allen, Zhen Hu, Hanna True, Jin Cho, Austin Harris, Nancy Fell, Mina Sartipi

**Affiliations:** ^1^Department of Computer Science and Engineering, University of Tennessee at Chattanooga, Chattanooga, TN 37403, USA; ^2^Department of Physical Therapy, University of Tennessee at Chattanooga, Chattanooga, TN 37403, USA

## Abstract

Falls are common and dangerous for survivors of stroke at all stages of recovery. The widespread need to assess fall risk in real time for individuals after stroke has generated emerging requests for a reliable, inexpensive, quantifiable, and remote clinical measure/tool. In order to meet these requests, we explore the Functional Reach Test (FRT) for real-time fall risk assessment and implement the FRT function in* mStroke*, a real-time and automatic mobile health system for poststroke recovery and rehabilitation.* mStroke* is designed, developed, and delivered as an Application (App) running on a hardware platform consisting of an iPad and one or two wireless body motion sensors based on different mobile health functions. The FRT function in* mStroke* is extensively tested on healthy human subjects to verify its concept and feasibility. Preliminary performance will be presented to justify the further exploration of the FRT function in* mStroke* through clinical trials on individuals after stroke, which may guide its ubiquitous exploitation in the near future.

## 1. Introduction

Falls are common for survivors of stroke at all stages of recovery [1–4]. Community-dwelling individuals with chronic stroke have the highest fall incidence at 46% [4]. Consequently, hip fractures are four-times more likely to occur in poststroke survivors compared to the general elderly population [5]. Falls also result in progressive activity and participation limitations, increased dependence, increased fear of falling, and depression [6]. Additionally, falls lead to significantly more stress for the caregivers of poststroke individuals [6, 7].

Fall prevention strategies are most effective if the person at risk can be assessed/identified before injury occurs [8–10]. There are several clinical tools that accurately assess functional parameters associated with standing balance and predict fall risk in individuals after stroke. Relevant clinical tools include Berg Balance Scale (BBS), Timed Up and Go (TUG) test, Computerized Dynamic Posturography (CDP) and force plates, and the FRT [11–15]. The BBS applies an ordinal rating scale to 14 functional movements [13]. The TUG is a functional walking test which measures task completion time [14, 16]. The CDP and force plates measure an individual's Center Of Pressure (COP) and COP correlates with poor balance and increased fall risk [15, 17, 18]. The previously listed clinical tools may require clinician administration and/or expensive or immobile equipment. Hence, they are suitable for clinical use but cannot longitudinally monitor community-dwelling individuals without the presence of a clinician and/or expensive equipment.

The application of accelerometer and gyroscope has been studied to quantitatively assess standing balance [18, 19]. These studies demonstrate the usefulness of motion sensors in functional balance measurement. However, both studies focus on improving the clinician's measurement sensitivity rather than producing a remote measurement system for mobile health. Methods in these studies cannot be applied at home without the presence of a clinician, due to test complexity (4-step and 6-step, respectively) and requirement (e.g., the user's eyes to be closed).

In this paper, we explore wearable technologies (i.e., real-time motion sensing) to assess fall risk using the FRT. The FRT is a quick single-task dynamic test defined as* the maximal distance one can reach forward beyond arm's length, while maintaining a fixed base of support in the standing position* [11]. Importantly, it has a modified version for sitting balance, that is, the Modified FRT, which we anticipate that it will prove useful for the sitting measurement development [20]. The FRT was developed by Duncan et al. in 1990 as a ratio measurement scale to determine anterior limits of standing balance in the elderly population [11]. Since its inception, this measure has been proven a valid and reliable test for identifying deficits in balance for stroke survivors and a powerful predictor of fall risk compared to other more time-consuming clinical functional measures [11, 21]. Specifically speaking, the FRT estimates how far the user can reach forward without taking steps [11]. The norms of reach distance for men and women of different ages are summarized in Table 1 [11]. Based on the reach distance in the FRT, a person at a high risk of falling (i.e., positive test) can be identified [9]:

- A negative test is considered for a forward reach of greater than 25.40 cm.
- A reach of less than 15.24 cm is found to be associated with a four times greater risk for falls during the following 6 months.
- A reach within 15.24–25.40 cm is found to be associated with a two times greater risk for falls during the following 6 months.

The real-time FRT is one of the functions in our proposed* mStroke*, a real-time and automatic mobile health system, which can also evaluate motor control and estimate gait speed of patients after stroke. Here, we focus on the FRT function in* mStroke* and address three complimentary problems: (i) designing signal processing algorithms that can accurately and faithfully estimate reach distance in FRT, (ii) implementing an interactive user-friendly App running on our hardware platform, and (iii) evaluating the usability and reliability of the FRT function in* mStroke* on healthy adult subjects.

Once the FRT function in* mStroke* demonstrates its usability and reliability in a healthy adult population, further development and evaluation will be executed in poststroke individuals. Our ultimate goal is that individuals after stroke will easily perform a real-time fall risk assessment by taking advantage of this FRT function in the clinic (e.g., any acute care/postacute care/rehabilitation facility) and home, at any time as needed, without help from healthcare professionals. In other words, the FRT can be transitioned from the skilled clinical administration to the independent patient management.* mStroke*, including the FRT function, can promote pervasive, quantifiable, and continued monitoring of patients' behaviors and recoveries, which can support efficient and long-term stroke management well beyond the current acute clinic-based system.

## 2. Materials and Methods

### 2.1. Hardware and Its User Friendliness

**Figure 1:**
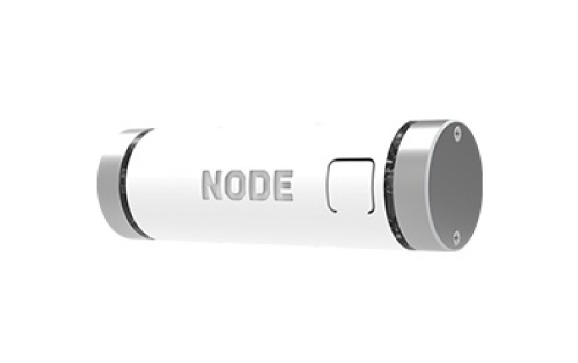
NODE.

Energy and latency are two major constraints on any wireless or mobile health device. We chose NODE, shown in Figure 1, as the wireless body sensor for* mStroke* [22]. This low-power and low-latency hand-held device is a new modular sensor platform that uses the Bluetooth Low Energy (BLE) protocol to communicate with a base station (e.g., smartphone, iPad, or computer). Multiple NODEs can connect with a single base station. The basic module of NODE is the MPU-9150, a 9-axis MotionTracking device manufactured by InvenSense, which essentially is an Inertial Motion Unit (IMU) containing accelerometer, gyroscope, and magnetometer [23]. The accelerometer can be programmed to have the full-scale range of ±2 g, ±4 g, ±8 g, or ±16 g and its sensitivity is ±1200 LSB/g [23]. The MPU-9150 is designed for the low-power, low-cost, and high-performance requirements of consumer electronics including wearable sensors [23]. NODE can send motion data to an iPad at up to 120 samples per second with a range of up to 50 m. NODE is a 25.4 mm diameter cylinder with a length of 83.8 mm and can be clipped to clothing. Each end of NODE can accept an additional interchangeable sensor unit. These sensor units can serve a variety of functions such as temperature, moisture level, oximeter, or ultrasound monitoring/measurement. For the purposes of this paper, we only employ the NODE with an IMU.

In recent years, the idea of employing sensors (e.g., accelerometer, gyroscope, magnetometer, and electromyography) to acquire human motion data for rehabilitation studies and practices has received considerable attention [18, 19]. Accelerometers measure acceleration vector; gyroscopes provide angular rotation rate; and magnetometers measure the strength and, in some cases, the direction of magnetic fields. A 9-axis sensor fusion of these three sensors allows* mStroke* to overcome the inherent flaws found in each individual motion sensor.

**Figure 2:**
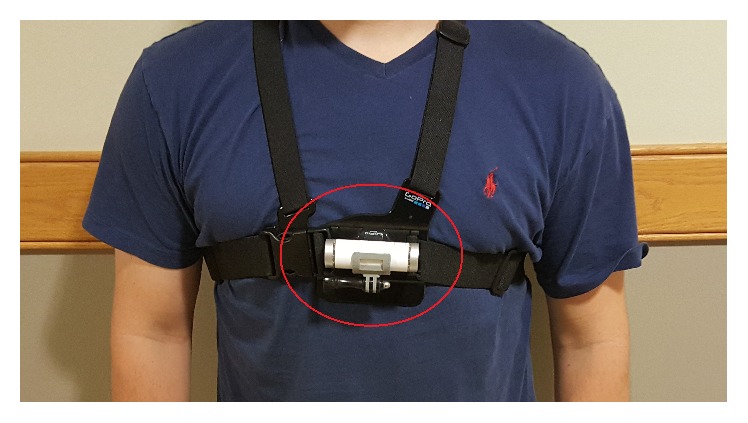
NODE on the chest for FRT.

In order to execute the FRT function, one NODE is worn via chest harness, which is shown in Figure 2. Donning and doffing the harness were tested by physical therapy students via skilled emulation. Results suggest translation of such a harness system to patient use. Additionally, if NODE is not worn correctly (e.g., NODE rotated or turned upside down), the App will send out a warning notification.

### 2.2. Software and Its User Friendliness

In terms of software functionality, the FRT function in* mStroke* includes fall risk assessment and error detection. Errors include faulty standing posture and falling. At the beginning of the FRT, the App asks the user to assume a comfortable, erect stance. The App notifies the user if the chest NODE detects an incorrect body posture. To account for individuals after stroke who may have impaired standing posture, trunk flexion up to 30° is acceptable [9]. The App then instructs the user to flex the shoulder of the dominant upper extremity (i.e., the lesser affected upper extremity in survivors after stroke) to approximately 90°. When the arm is properly positioned, the user will reach forward as far as possible without taking a step. Finally, the FRT distance is estimated based on our proposed algorithm.

The FRT function in* mStroke* is personalized for each individual user by inputting the user's trunk length, shoulder width, and thigh length into the App before the FRT is initiated. After the algorithm estimates the FRT distance, the result is announced to the user in real time based on established FRT norms (Table 1). To ensure safety,* mStroke* is equipped with a fall detection algorithm and can be programmed to provide automatic emergency medical service notification in case of a fall. For this purpose, we have implemented the 3-step fall detection algorithm proposed by Li et al. [24].

### 2.3. The FRT Distance Estimation

#### 2.3.1. Angle Estimation

There are accelerometer, gyroscope, and magnetometer in the NODE IMU. We exploit quaternion calculated from readings of these three sensors for accurate angle estimation. A quaternion is a four-dimensional complex number that can be used to represent the orientation of a rigid body in a three-dimensional space [25]. In quaternion representation, q^BA describes the orientation of frame *B* relative to frame *A* [25]. Any orientation of frame *B* relative to frame *A* can be achieved through a rotation of angle *θ* around axis r^ A defined in frame *A* [25]. The quaternion q^BA describing this orientation is defined as follows:

(1) q^BAq0q1q2q3=cos⁡θ2−rXsin⁡θ2−rYsin⁡θ2−rZsin⁡θ2,

where *r*_*X*_, *r*_*Y*_, and *r*_*Z*_ define the components of the unit vector r^ A in *x*-, *y*-, and *z*-axes of frame *A*, respectively [25].

Assume the reference quaternion is q^BA; the current quaternion is q^CA; and the orientation between q^BA and q^CA is q^CB. Then the relationship among q^CA, q^CB, and q^BA can be represented as follows [25]:

(2) q^CA=q^CB⊗q^BA,

where ⊗ denotes the quaternion product which can be determined using the Hamilton rule [25]:

(3) a⊗ba0a1a2a3⊗b0b1b2b3=a0b0−a1b1−a2b2−a3b3a0b1+a1b0+a2b3−a3b2a0b2−a1b3+a2b0+a3b1a0b3+a1b2−a2b1+a3b0T.

The quaternion conjugate, denoted by *∗*, can be used to swap the relative frames described by an orientation [25]:

(4) q^∗BA=q^AB=q0−q1−q2−q3.

Based on (2) and (4), we can easily get the following:

(5) q^CB=q^CA⊗q^AB=q^CA⊗q^∗BA.

A three-dimensional vector can be rotated by a quaternion [25]. If ${\;}^{B}\hat{v}$ and ${\;}^{C}\hat{u}$ are the same vector described in frame *B* and frame *C*, respectively, then we get the following:

(6) u^ C=q^CB⊗v^ B⊗q^∗CB,

where v^ B and u^ C contain 0 as the first element to make them four-dimensional vectors [25].

Angle *θ* corresponding to such a rotation can be obtained from the angle of two vectors, that is, u^ C and v^ C, where v^ B and v^ C have the same mathematical expressions but represent different vectors:

(7) θ=arccos⁡u^ C·v^ Cu^ C2v^ C2.

**Figure 3:**
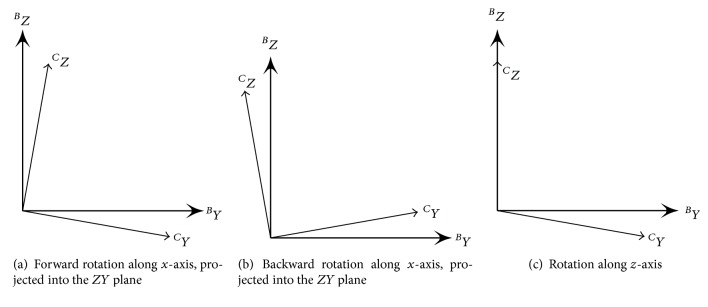
Rotation from frame *B* to frame *C*.

However, *θ* calculated based on (6) and (7) has two problems for our practical implementation. One problem is that *θ* is always positive and the other problem is that *θ* can be in any rotation direction. We will explain these two problems using illustrative examples shown in Figure 3. Figures 3(a) and 3(b) represent forward rotation and back rotation from frame *B* to frame *C* along *X*-axis, respectively.  Figure 3(c) represents a rotation along *Z*-axis. Assume the absolute values of angles for all rotations are *θ*, (0° < *θ* < 180°). Based on (6), u^ C corresponds to _ _^*B*^ *Y* and v^ C corresponds to _ _^*C*^ *Y*. Furthermore, if (7) is applied, we will get the following:

(8) θYa→ BY C=θY Bb→Y C=θ,

where $\underset{\to }{a}$ and $\underset{\to }{b}$ denote the rotations shown in Figures 3(a) and 3(b), respectively. Thus, we cannot differentiate forward rotation and backward rotation from θYa→ BY C and θY Bb→Y C. Taking Figure 3(c) into account, if we are only interested in a rotation in the *ZY* plane of frame *B*, we should get 0° for the angle of such a rotation. However, we still get *θ* instead of 0° by using (6) and (7).

In order to address these two problems, we propose the following solution to obtain *θ* as expected. In addition to (6), we apply the second vector rotation as follows:

(9) t^ C=q^CB⊗s^ B⊗q^CB∗.

Assume t^ C and s^ B correspond to _ _^*B*^ *Z* in frame *C* and frame *B*, respectively. Then, we find the angle between _ _^*B*^ *Z* and _ _^*C*^ *Y* by slightly updating (7) as follows:

(10) θ=arccos⁡t^ C·v^ Ct^ C2v^ C2.

In this way,

(11) θY Ba,b,c→Y C=θZ Ba,b,c→Y C−90°.

In summary, the proposed solution can address the aforementioned problems illustrated in Figure 3:

(i) In Figure 3(a) for forward rotation, θZ Ba→Y C=90°+θ and θY Ba→Y C=θ.
(ii) In Figure 3(b) for backward rotation, θZ Bb→Y C=90°-θ and θY Bb→Y C=-θ.
(iii) In Figure 3(c) for rotation along *Z*-axis, θZ Bc→Y C=90° and θY Bc→Y C=0°, which means the angle of such a rotation projected in the *ZY* plane of frame *B* will be 0°.

#### 2.3.2. Functional Reach due to Trunk Flexion

Based on the clinical observation, the reach in the FRT is mainly executed through trunk flexion. If we can estimate trunk flexion angle based on the proposed algorithm presented in Section 2.3.1, we can calculate the corresponding reach distance *d*_1_ according to trigonometric function as follows:

(12) $$\begin{array}{c} d_{1}=L_{\text{trunk}}\sin\left(\;\theta_{\text{trunk flexion}}\;\right), \end{array}$$

where *L*_trunk_ denotes trunk length measured manually and *θ*_trunk  flexion_ denotes trunk flexion angle estimated automatically by* mStroke*. The IMU in the chest NODE provides the necessary quaternion information to estimate trunk flexion angle.

#### 2.3.3. Effect of Torso Twist

*d*_1_ only considers the functional reach due to trunk flexion. However, the human body is not strictly a rigid body. When the FRT is performed, there is an inevitable torso twist. The torso twist will also contribute to the functional reach. With the 3-axis IMU in the chest NODE, we can estimate torso twist angle simultaneously with trunk flexion angle. Thus, *d*_1_ can be updated as *d*_2_:

(13) $$\begin{array}{c} d_{2}=d_{1}+W_{\text{shoulder}}\sin\left(\;\theta_{\text{torso twist}}\;\right), \end{array}$$

where *W*_shoulder_ denotes shoulder width measured manually and *θ*_torso  twist_ denotes torso twist angle estimated automatically by* mStroke*.

#### 2.3.4. Effect of Thigh Movement

When an individual performs the FRT, the lower body does not remain perpendicular to the ground. The lower body may sometimes displace backward to keep the person's center of mass within his/her base of support. Any lower body deviation from the original vertical position may affect the FRT result. Hence, we need to explicitly consider such an effect. It is impossible for the IMU in the chest NODE to capture the lower body movement in the FRT. Thus, we exploit a second NODE on the thigh to estimate thigh movement angle. Based on this angle, we can quantify the lower body movement which contributes to the functional reach as *L*_thigh_sin⁡(*θ*_thigh  movement_) where *L*_thigh_ denotes thigh length measured manually and *θ*_thigh  movement_ denotes thigh movement angle estimated automatically by* mStroke*. Eventually, we propose the third reach distance measure *d*_3_ as follows:

(14) $$\begin{array}{c} d_{3}=d_{2}+L_{\text{thigh}}\sin\left(\;\theta_{\text{thigh movement}}\;\right). \end{array}$$

## 3. Results and Discussion

### 3.1. The FRT Reliability Method

The FRT reliability study was conducted on healthy adult subjects in a research setting with appropriate IRB approval. Subjects provided informed consent prior to participation. Age and gender were recorded as subject demographics. Due to a sample of convenience, healthy college students, most of our subjects have a normal body mass index. Any outliers would be considered overweight, not obese.

For each subject, trunk length, shoulder width, and thigh length were measured manually and entered into the App before the FRT was initiated. A measuring tape was secured to the wall at the shoulder height of each subject.

With clinician cueing, the subject was positioned standing next to the wall-mounted measuring tape so that his/her reach would not exceed the length of the measuring tape. The subject was then instructed to raise his/her upper extremity to 90°. The starting position was assessed by the clinician at the subject's distal third phalange. The subject was subsequently asked to reach forward as far as comfortably possible, without taking a step. At the peak of the subject's reach, the clinician marked the reach end. The absolute distance between these two marked positions on the measuring tape was used as the comparison benchmark for the* mStroke* estimated reach distance. We tested the FRT function in* mStroke* on two groups of subjects to verify its performance. Each subject performed the FRT five times.

### 3.2. The FRT Performance

**Figure 4:**
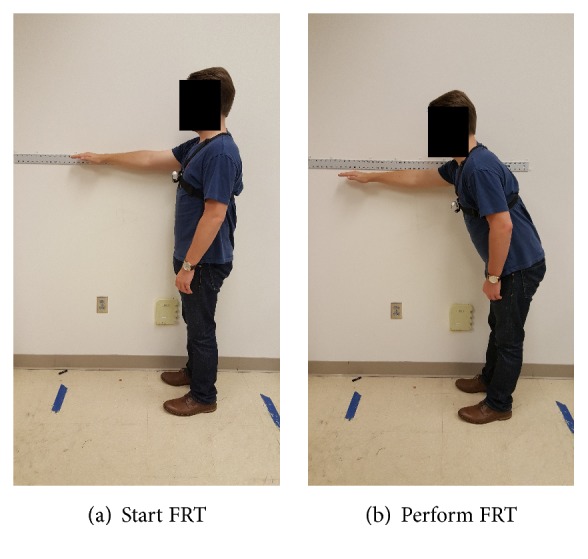
A subject performing the FRT wearing a single chest NODE, Group 1.

**Figure 5:**
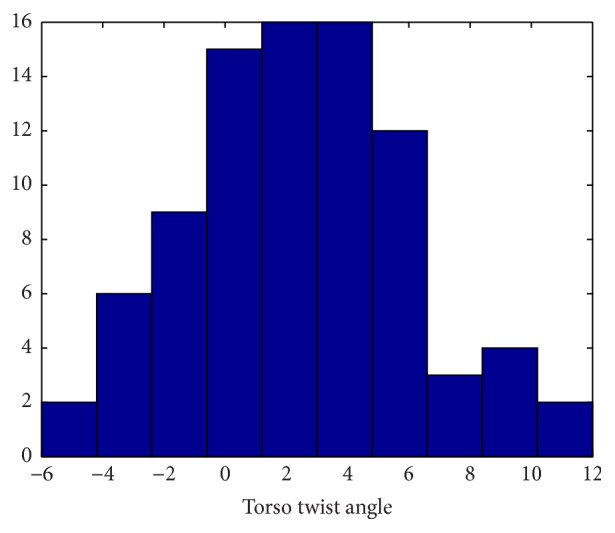
Histogram plot of torso twist angle (°), Group 1.

**Figure 6:**
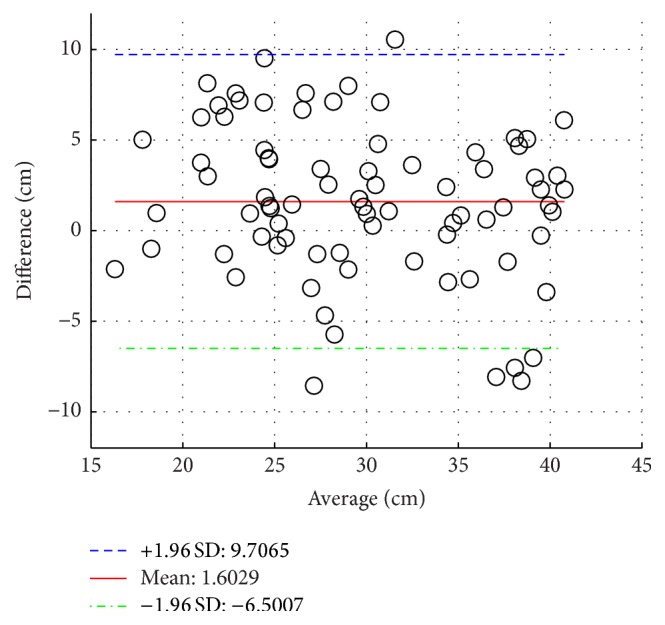
Bland Altman plot between *d*_PT_ and *d*_1_, Group 1.

**Figure 7:**
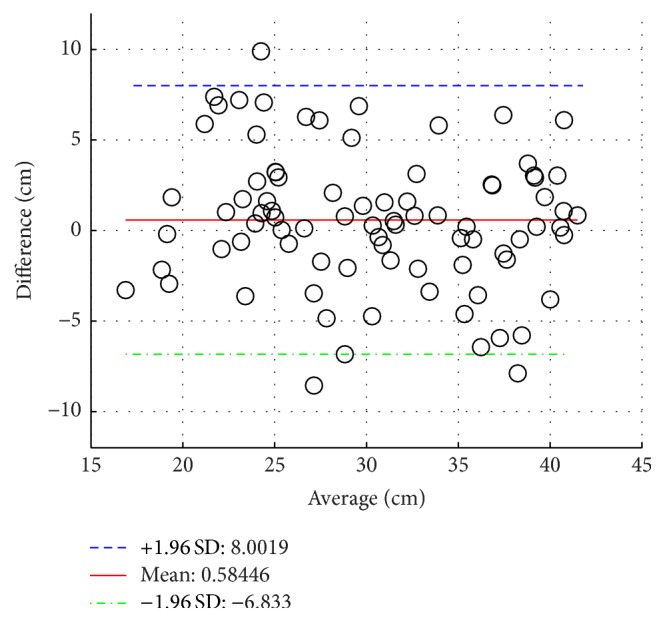
Bland Altman plots between *d*_PT_ and *d*_2_, Group 1.

Group 1 includes 17 healthy adult subjects.  Table 2 presents Group 1 demographic data. One NODE (positioned on the chest) is used in Group 1 to estimate trunk flexion and torso twist angles, as shown in Figure 4. The histogram of torso twist angles is presented in Figure 5. It can be easily observed from Figure 5 that most of torso twist angles are not equal to 0°, which will bring nontrivial effect on the functional reach result. The performances of reach distance estimation in terms of Mean Absolute Error (MAE) and correlation coefficient are given in Table 3 where *d*_PT_ denotes the reach distance manually measured by a clinician and serves as the performance benchmark for the FRT function in* mStroke*. *d*_1_ and *d*_2_ are described in (12) of Section 2.3.2 and (13) of Section 2.3.3, respectively. With consideration of MAE, *d*_2_ outperforms *d*_1_ by 17.0%. Bland Altman plots between *d*_PT_ and *d*_1_ as well as between *d*_PT_ and *d*_2_ are shown in Figures 6 and 7, respectively. Mean of differences shows the bias/discrepancy between the measurement and the benchmark. +1.96 Standard Deviation (SD) of differences and −1.96 SD of differences give the range of 95% limits of agreement. The most of the differences fall within such a range.

**Figure 8:**
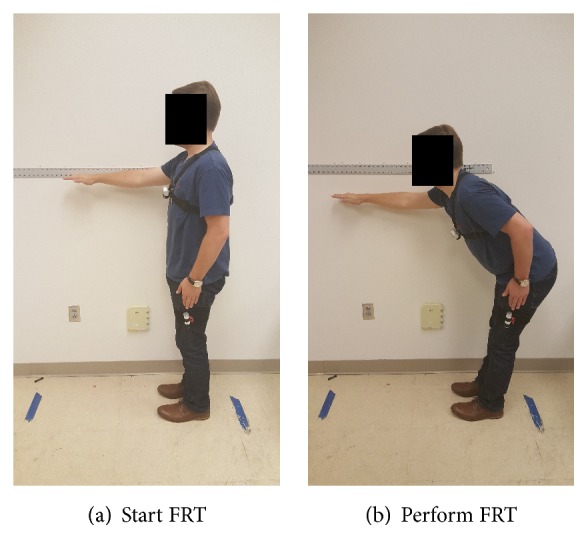
A subject performing the FRT using two NODEs on the chest and the left thigh, respectively, Group 2.

**Figure 9:**
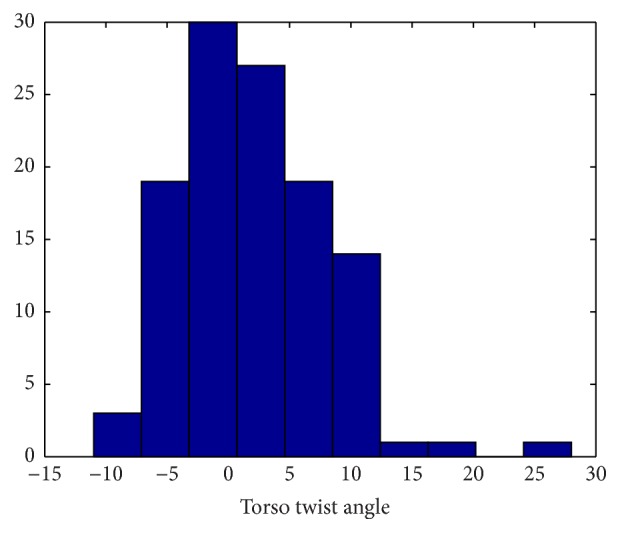
Histogram plot of torso twist angle (°), Group 2.

**Figure 10:**
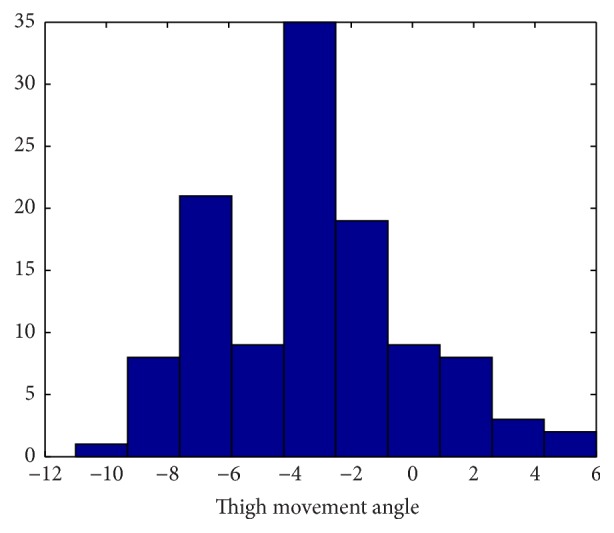
Histogram plot of thigh movement angle (°), Group 2.

Group 2 includes 23 healthy adult subjects with demographics shown in Table 4. In contrast to Group 1, two NODEs are used in Group 2 to estimate trunk flexion, torso twist, and thigh movement angles (see Figure 8). The histograms of torso twist and thigh movement angles are shown in Figures 9 and 10, respectively. Both figures clearly show that nonzero angles for torso twist and thigh movement dominate the tests. The corresponding performances are shown in Table 5. *d*_3_ is described in (14) of Section 2.3.4. With consideration of MAE, *d*_2_ outperforms *d*_1_ by 1.62% and *d*_3_ further improves the performance by 17.6%.

While the experimental results are promising, there is still room for performance improvement. Our studies clearly suggest that more motion sensors (e.g., sensor on the shoulder or arm) should be considered to further improve the performance of the FRT function in* mStroke* by capturing more detailed body movements in the FRT exercise.

## 4. Conclusions

We have designed and developed a mobile health system (i.e.,* mStroke*) which can perform the FRT, an accurate single-task clinical tool, for real-time fall risk assessment. Three different reach distance measures (i.e., *d*_1_, *d*_2_, and *d*_3_) have been given. The reliability of* mStroke*'s FRT function has been tested on two groups of healthy adult subjects. The experimental results verify its concept and feasibility. A clinical trial on individuals after stroke is the next step for the further development of the FRT function in* mStroke*.

## Figures and Tables

**Table 1:** Functional reach norms.

Age	Men	Women
20–40	42.49 cm	37.19 cm
41–69	38.05 cm	35.08 cm
70–87	33.43 cm	26.59 cm

**Table 2:** Subject demographics, Group 1.

Gender	Number	Age (mean)
Female	10	23.6
Male	7	23.9

Total	17	23.7

**Table 3:** FRT results, Group 1.

	*d*_PT_ versus *d*_1_	*d*_PT_ versus *d*_2_
MAE	3.53 cm	2.93 cm
Correlation coefficient	0.83	0.85

**Table 4:** Subject demographics, Group 2.

Gender	Number	Age (mean)
Female	15	26.3
Male	8	26.9

Total	23	26.5

**Table 5:** FRT results, Group 2.

	*d*_PT_ versus *d*_1_	*d*_PT_ versus *d*_2_	*d*_PT_ versus *d*_3_
MAE	4.32 cm	4.25 cm	3.50 cm
Correlation coefficient	0.61	0.61	0.70
